# Comparative assessment of KDIGO and CTCAE criteria for detecting renal function deterioration after peptide receptor radionuclide therapy

**DOI:** 10.1007/s00259-026-08126-4

**Published:** 2026-08-12

**Authors:** Sophie C. Siegmund, Mohamed Salem, Elma Dema, Liam Widjaja, Franz Josef Gildehaus, Christine Spitzweg, Christoph J. Auernhammer, Matthias Brendel, Rudolf A. Werner

**Affiliations:** 1 Department of Nuclear Medicine, LMU University Hospital, LMU Medizin, LMU Munich, Marchioninistr. 15, Munich, 81377 Germany; 2 Interfaculty Centre for Endocrine and Cardiovascular Disease Network Modelling and Clinical Transfer (ICONLMU), LMU University Hospital, LMU Medizin, LMU Munich, Munich, Germany; 3 Department of Internal Medicine IV, LMU University Hospital, LMU Medizin, LMU Munich, Munich, Germany; 4 ENETS Certified Center of Excellence), Interdisciplinary Center of Neuroendocrine Tumors of the GastroEnteroPancreatic System (GEPNET- KUM, LMU University Hospital, LMU Medizin, LMU Munich, Munich, Germany; 5 German Center for Neurodegenerative Diseases (DZNE), Munich, Germany; 6 Munich Cluster for Systems Neurology (SyNergy), Munich, Germany

**Keywords:** PRRT, Nephrotoxicity, CTCAE, KDIGO, EGFR, EGFR_cr_ slope

## Abstract

**Purpose:**

Renal toxicity after peptide receptor radionuclide therapy (PRRT) is commonly assessed using Common Terminology Criteria for Adverse Events (CTCAE), whereas nephrological classification relies on Kidney Disease: Improving Global Outcomes (KDIGO)-based renal function categories. We compared CTCAE- and KDIGO-derived eGFR_cr_-based definitions of renal function deterioration after PRRT and evaluated their predictive value for subsequent long-term renal outcome.

**Methods:**

149 gastroenteropancreatic neuroendocrine tumor patients scheduled for PRRT with renal follow-up for at least 12 months after treatment were included. Kidney function was assessed at baseline (BL), after the last cycle (LC) and last available follow-up (FU). Analyzing the time interval from BL to LC, estimated glomerular filtration rate (eGFR_cr_)-based KDIGO and CTCAE classifications along with risk factors for recategorization were compared. Clinically relevant KDIGO worsening was defined according to KDIGO 2012 (eGFR_cr_ category worsening with an additional eGFR_cr_ decline ≥ 25%).

**Results:**

Median eGFR_cr_ declined from 87 to 68 mL/min/1.73 m² over a median follow-up of 2.2 years (*p* < 0.0001). From BL to FU, clinically relevant KDIGO worsening was substantially discordant from CTCAE eGFR_cr_ grading (KDIGO-/CTCAE+: 21 vs. KDIGO+/CTCAE-: 2; *p* < 0.0001). In multivariable analysis, age (*p* = 0.0125) and cumulative activity (*p* = 0.0205) were associated with clinically relevant KDIGO worsening (CTCAE eGFR_cr_ worsening: age, *p* < 0.0209). Early CTCAE eGFR_cr_ worsening during BL to LC predicted subsequent annualized eGFR decline until further FU (β − 6.82; 95% CI − 10.06 to − 3.57; *p* < 0.0001). Both clinically relevant KDIGO eGFR_cr_ worsening and absolute eGFR_cr_ at LC, however, failed to reach significance for long-term outcome prediction of renal function (*p* > 0.05, each).

**Conclusion:**

For evaluation of renal safety under PRRT, clinically relevant KDIGO deterioration and CTCAE grading are not interchangeable. Clinically relevant KDIGO-derived eGFR_cr_ worsening represents a stricter filtration-based endpoint, whereas early CTCAE eGFR_cr_ worsening may help identify patients requiring closer post-treatment renal surveillance. Prospective studies integrating serial filtration markers, urine biomarkers, measured GFR, and individualized kidney dosimetry are warranted, especially in light of current ongoing trials investigating PRRT being moved to an earlier stage in the treatment course.

**Supplementary Information:**

The online version contains supplementary material available at https://doi.org/10.1007/s00259-026-08126-4.

## Introduction

Peptide receptor radionuclide therapy (PRRT) is an established treatment option for patients with gastroenteropancreatic neuroendocrine tumors (GEP-NET) [1]. Although PRRT is generally well tolerated, the kidneys remain a significant organ at risk due to the partial reabsorption and retention of radiolabeled somatostatin analogues in the proximal tubules [2, 3]. Amino acid co-infusion has substantially reduced clinically severe nephrotoxicity, but gradual renal function decline may still occur during long-term follow-up (FU) [4, 5]. This phenomenon may become even more relevant in light of currently recruiting trials which aim to move PRRT earlier in the treatment course [6].

In this regard, renal toxicity subsequent to PRRT is commonly reported using the Common Terminology Criteria for Adverse Events (CTCAE), which provides standardized oncological toxicity reporting [7]. Conversely, nephrological disease progression is predominantly based on estimated glomerular filtration rate (eGFR) categories as defined by Kidney Disease: Improving Global Outcomes (KDIGO) [8, 9]. However, whether early CTCAE or KDIGO classification changes are informative for subsequent long-term eGFR decline after PRRT remains insufficiently defined. Those framework-based reassignments of renal function from baseline (BL) to last cycle (LC), however, may be of particular clinical relevance, as PRRT is mainly associated with long-term kidney damage after completion of treatment [10].

**Fig. 1:**
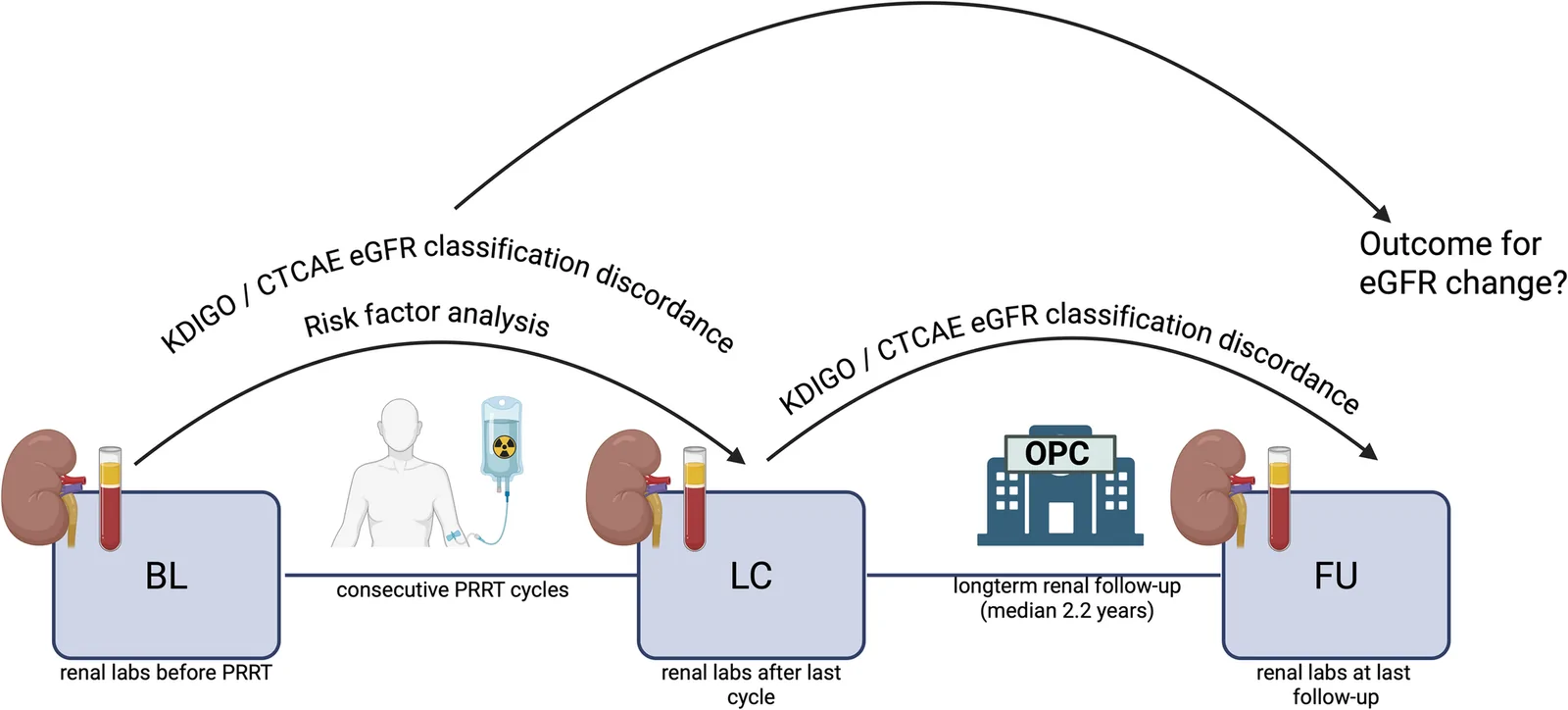
Study timeline and renal outcome analysis workflow. Renal laboratory parameters were assessed at baseline (BL), after the last cycle of the initial consecutive PRRT course (LC), and at last follow-up (FU). Longitudinal renal function was evaluated across all three time points. KDIGO and CTCAE eGFR classification changes were compared from BL to FU and from LC to FU. Early classification changes from BL to LC were further assessed as predictors of subsequent annualized eGFR slope from LC to FU. Created in BioRender. Kunte, S. (2026) https://BioRender.com/6uw76ly

The present study therefore compared KDIGO and CTCAE eGFR-based renal toxicity classification changes from BL to LC in a GEP-NET cohort treated with PRRT. In addition, we assessed longitudinal eGFR_cr_ (eGFR creatinine) change, evaluated clinical risk factors associated with observed renal deterioration under PRRT, and tested whether early CTCAE or KDIGO classification changes predicted subsequent eGFR_cr_ decline from LC to last follow-up (FU). The study design is shown in Fig. 1.

## Methods

### Study population

This retrospective study comprised patients with histologically confirmed GEP-NET treated with a minimum of one PRRT cycle. Additionally, renal follow-up data had to be available for at least 12 months after the LC of the initial consecutive PRRT treatment course. The initial treatment course was defined as a maximum of four consecutive PRRT cycles. Treatment was conducted at our ENETS-certified Center of Excellence for GEP-NET. The study was approved by the Ethics Committee of the Ludwig-Maximilians-University Munich (IRB #25–0233, #25–0131).

### PRRT

[^177^Lu]Lu-Oxodotreotid (Lutathera^®^) was obtained from Novartis Radiopharmaceuticals GmbH (Nuremberg, Germany). No-carrier added [^177^Lu]Lutetium was supplied by Isotope Technologies Munich S.E. (EndolucinBeta^®^, Garching, Germany). DOTA-3-iodo-Tyr3-octreotate was provided by att/Scintomics GmbH (Fürstenfeldbruck, Germany). PRRT was administered intravenously. For nephroprotection, amino acids (2.5% Lysine and 2.5% Arginine) were administered 30 min prior to the administration of the radiopharmaceutical following current guidelines [11].

### Renal laboratory assessment

Renal function was assessed at three predefined time points: BL before PRRT, after the completion of the LC of the initial consecutive PRRT course, and at the last available renal FU. eGFR creatinine (eGFR_cr_) was calculated using the CKD-EPI 2021 formula [12]. Serum creatinine and serum urea were analyzed as additional renal laboratory parameters. The absolute values at each time point and relative changes from BL to FU were calculated. The annualized eGFR_cr_ slope was defined as the difference between eGFR_cr_ at FU and BL divided by the renal follow-up duration in years [13]. For prediction analyses, subsequent annualized eGFR_cr_ slope from LC to FU was defined as the difference between eGFR_cr_ at FU and LC divided by the time interval from LC to FU in years.

In patients with available cystatin C measurements at BL and LC, additional exploratory analyses were performed. Cystatin C-based eGFR (eGFR_cys_) was calculated using the CAPA equation, and combined creatinine–cystatin C-based eGFR (eGFR_cr−cys_) was calculated according to the respective CKD-EPI Eqs. [14, 15]. Paired BL-to-LC cystatin-based analyses were restricted to patients with cystatin C measurements available at both time points.

### Nephrotoxicity classification

Renal function was classified according to KDIGO eGFR_cr_ categories and defined as follows: G1 (eGFR ≥ 90 mL/min/1.73 m²), G2 (60–89), G3a (45–59), G3b (30–44), G4 (15–29) and G5 (< 15) [8, 9]. CTCAE eGFR_cr_-based toxicity grading were evaluated according to CTCAE v5.0: grade 1 eGFR_cr_ or CrCl (creatinine clearance) < LLN − 60 ml/min/1.73 m^2^), grade 2 (eGFR_cr_ or CrCl 59–30 ml/min/1.73 m2), grade 3 (eGFR_cr_ or CrCl 29–15 ml/min/1.73 m^2^), grade 4 (eGFR_cr_ or CrCl < 15 ml/min/1.73 m^2^; dialysis or renal transplant indicated) [7].

The occurrence of *any* KDIGO eGFR_cr_ worsening was defined as decline of at least one KDIGO eGFR_cr_ category in comparison with the corresponding reference time point. *Clinically relevant* KDIGO eGFR_cr_ worsening was evaluated as a stricter renal deterioration endpoint and defined as a decline in KDIGO category accompanied by a 25% or greater drop in eGFR_cr_ from BL [9].

CTCAE worsening was defined as an increase in CTCAE grade relative to the respective reference time point. Analyses were conducted to evaluate renal deterioration from BL to FU and LC to FU (Fig. 1).

Additional sensitivity analyses were performed using a KDIGO 2024-derived threshold of > 20% relative eGFR_cr_ decline [8].

### Statistical analysis

Continuous variables are presented as median with interquartile range (IQR) or mean ± standard deviation, as appropriate. Categorical variables are reported as counts and percentages. Differences in parameters across BL, LC and FU were assessed using the Friedman test for repeated measures in patients with complete data at all three time points. Post-hoc pairwise comparisons were performed using Dunn’s multiple comparisons test. Paired BL-to-LC comparisons of cystatin-based eGFR estimates were performed using the Wilcoxon matched-pairs signed-rank test. Differences between eGFR_cr_, eGFR_cys_ and eGFR_cr/cys_ at BL and LC were assessed using the Friedman test with Dunn’s multiple comparisons test.

The agreement between KDIGO and CTCAE grading systems was assessed using cross-tabulation and weighted Cohen’s kappa for ordinal classifications (GraphPad QuickCalcs). Paired discordance between binary toxicity endpoints was assessed using McNemar tests. The annualized eGFR_cr_ slope was then compared descriptively between toxicity groups and across KDIGO/CTCAE discordance groups. Differences across discordance groups were analyzed using the Kruskal–Wallis test with subsequent Dunn’s multiple comparisons test. To assess whether early renal toxicity classification predicted subsequent renal outcome, early worsening from BL to LC (any KDIGO eGFR_cr_ worsening, clinically relevant KDIGO eGFR_cr_ worsening, CTCAE eGFR_cr_ worsening, KDIGO2024 eGFR_cr_ worsening) were tested as predictors of annualized eGFR slope from the LC to FU using univariable linear regression. In exploratory cystatin-based analyses, early eGFR_cys_ loss and eGFR_cr/cys_ loss from BL to LC were also tested as predictors of subsequent annualized eGFR_cr_ slope from LC to FU.

Univariable logistic regression was utilized to evaluate the associations between risk factors and clinically relevant KDIGO eGFR_cr_ worsening and CTCAE eGFR_cr_ worsening from BL to LC. Significantly associated variables in univariable logistic regression were entered into a multivariable logistic regression model. Risk factors included age, cumulative administered activity, sex, arterial hypertension and diabetes mellitus. The results of the regression analysis are reported in the form of odds ratios (OR) for logistic regression and beta coefficients for linear regression. Statistical significance was defined as two-sided *p* < 0.05. The analyses were conducted using GraphPad Prism version 10.6.1 (GraphPad Software, La Jolla, CA, USA).

## Results

### Patients’ characteristics

**Fig. 2:**
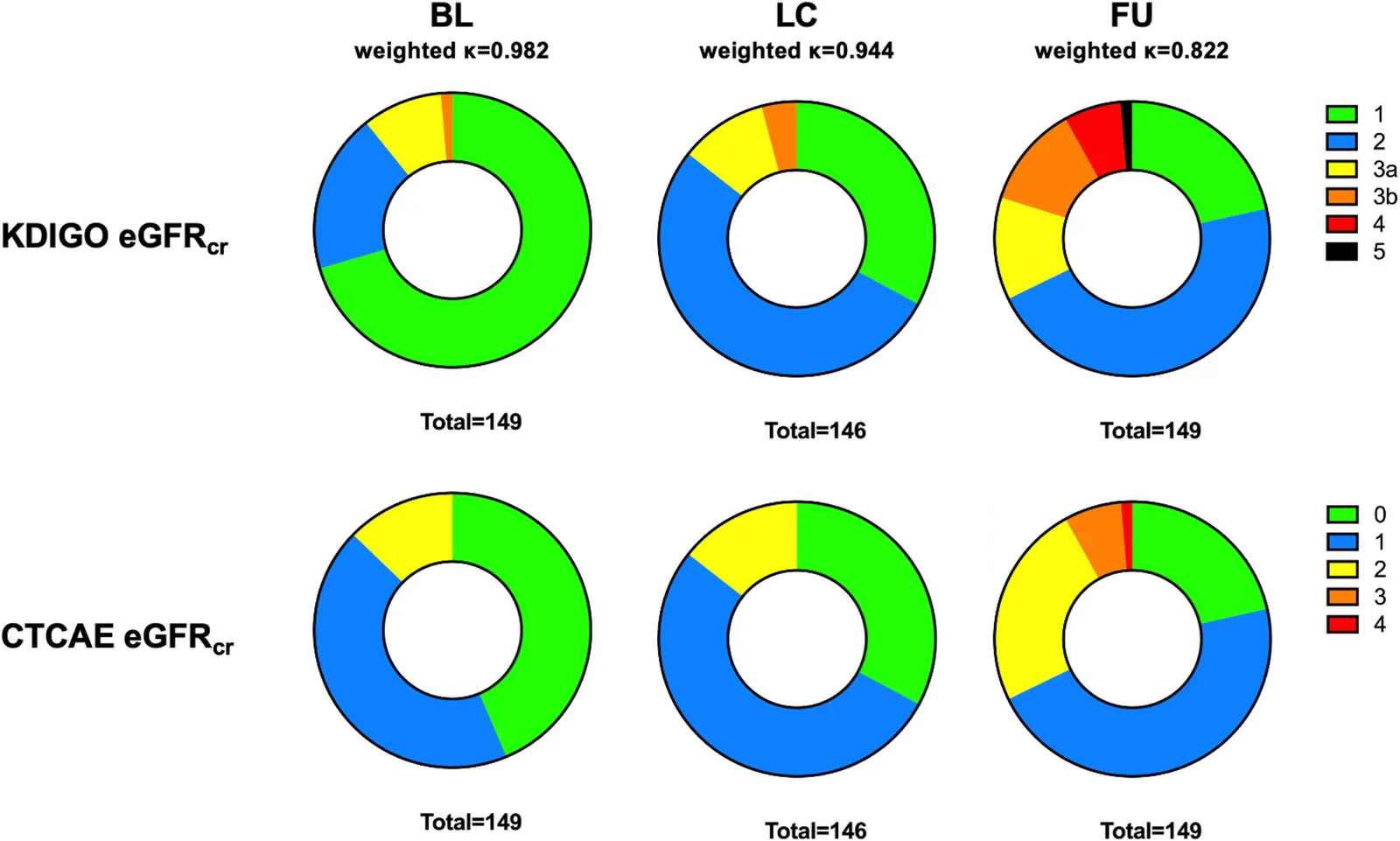
Distribution of KDIGO and CTCAE renal function classifications. Doughnut charts show the proportional distribution of KDIGO eGFR_cr_ categories and CTCAE eGFR_cr_ grades at baseline (BL), after the last PRRT cycle (LC), and at last follow-up (FU). Total patient numbers are shown below each chart. Weighted Cohen’s kappa values are displayed for each time point. The underlying raw count data are provided in Supplementary Table 1. BL, baseline; LC, last PRRT cycle; FU, last follow-up; eGFRcr, creatinine-based estimated glomerular filtration rate; PRRT, peptide receptor radionuclide therapy; CTCAE, Common Terminology Criteria for Adverse Events

A total of 149 patients were included. The complete raw count distribution underlying KDIGO eGFR_cr_ categories and CTCAE eGFR_cr_ grades at all assessed time points is provided in Supplementary Table 1, while Fig. 2 summarizes the corresponding proportional distributions and cross-sectional agreement.

Mean age was 62 ± 12 years. 85/149 (57.0%) patients were male and 64/149 (43.0%) female. The median follow-up was 2.2 years (IQR 1.4–3.7; range 1.0–8.4 years). The median cumulative administered activity was 29.6 GBq (IQR 28.9–30.7 GBq).

### Renal function declines from BL to LC to FU

**Fig. 3:**
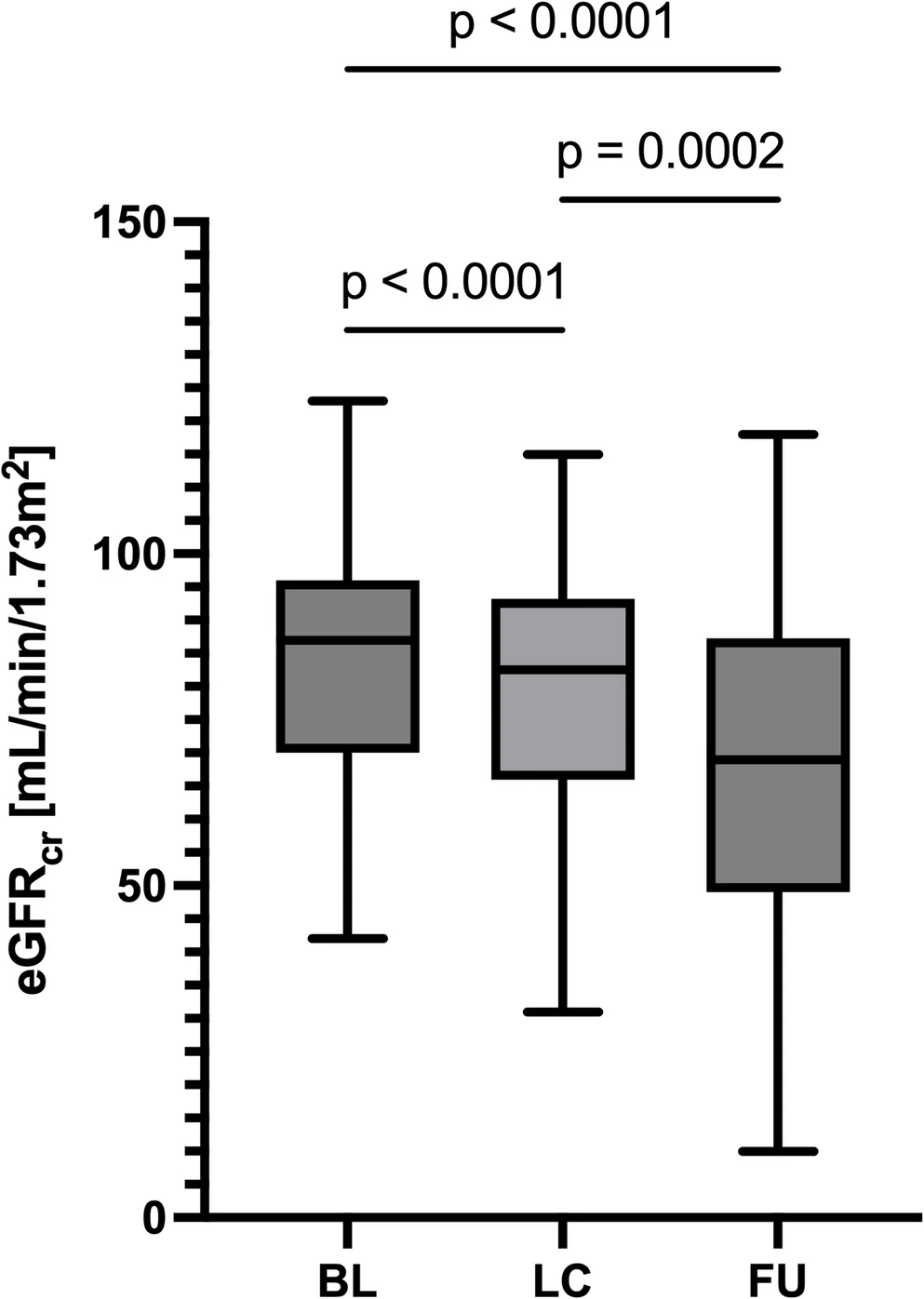
Longitudinal change in eGFR_cr_ across renal assessment time points. Box-and-whisker plots showing eGFR_cr_ at baseline (BL), after the last cycle of the initial PRRT course (LC), and at last follow-up (FU). The central line indicates the median, and whiskers indicate the minimum and maximum. P values for paired comparisons are displayed directly above the plots. Median eGFR decreased significantly from BL to LC and further from LC to FU. BL, baseline; LC, last PRRT cycle; FU, last follow-up; eGFRcr, creatinine-based estimated glomerular filtration rate; PRRT, peptide receptor radionuclide therapy

Renal function declined at every time point. Median eGFR_cr_ decreased from 87 mL/min/1.73 m² at BL to 83 mL/min/1.73 m² at LC and 68 mL/min/1.73 m² at FU (Friedman test *p* < 0.0001). Post-hoc testing showed significant differences between BL and LC (*p* < 0.0001), BL and FU (*p* < 0.0001), and LC and FU (*p* < 0.0002), see Fig. 3. The median creatinine increased from 0.9 mg/dL at BL to 1.1 mg/dL at FU (*p* < 0.0001), while median serum urea increased from 30.0 to 38.0 mg/dL (available in 133 patients; *p* < 0.0001). Median annualized eGFR_cr_ slope was − 4.5 mL/min/1.73 m²/year (IQR − 12.7 to − 0.8). The median relative eGFR_cr_ change from BL to FU was − 13.2% (IQR − 35.4 to − 2.7%), whereas median relative creatinine change was + 16.7% (IQR 0.0 to 39.3%; Table 1).

**Table 1:** Renal laboratory values

Parameter	BL	LC	FU
eGFR_cr_, mL/min/1.73 m²	87 (70–96)	83 (66–93)	68 (50–87)
Serum creatinine, mg/dL	0.9 (0.8–1.0)	0.9 (0.8–1.1)	1.1 (0.9–1.3)
Serum urea, mg/dL	30.0 (24–38)	33.0 (27–40)	38.0 (29–53)
eGFR_cr_ slope, mL/min/1.73 m²/year			−4.5 (− 12.7 to − 0.8)
Relative eGFR_cr_ BL→FU			−13.2% (− 35.4 to − 2.7)

Baseline (BL) laboratory values; Δ Delta; eGFR_cr_ estimated glomerular filtration rate creatinine; Follow-up (FU) laboratory values at the last follow-up visit; Last cycle (LC) laboratory values after the last PRRT cycle

### Clinically relevant KDIGO-derived eGFR_cr_ worsening is discordant from CTCAE eGFR_cr_ worsening despite high cross-sectional agreement

Agreement between KDIGO GFR categories and CTCAE GFR grades was very high at all assessed time points. Weighted Cohen’s kappa was 0.982 at baseline, 0.944 after the last PRRT cycle and 0.822 at last follow-up.

Any KDIGO eGFR_cr_ category worsening from BL to FU occurred in 72 (48.3%) patients, whereas CTCAE eGFR_cr_ worsening occurred in 67 (45.0%) patients. McNemar testing for any KDIGO eGFR_cr_ worsening versus CTCAE eGFR_cr_ worsening was not significant for BL to FU (*p* = 0.0625) or LC to FU (*p* = 0.0625). In contrast, clinically relevant KDIGO eGFR_cr_ worsening showed significant discordance in comparison with CTCAE eGFR_cr_ worsening at both intervals. For BL to FU, 46 patients were positive by both systems, 2 were KDIGO+/CTCAE−, 21 were KDIGO−/CTCAE + and 80 were negative by both systems (*p* < 0.0001), see Table 2. BL median eGFR_cr_ was significantly different for both discordance groups (KDIGO+/CTCAE−: 55 mL/min/1.73 m² vs. KDIGO−/CTCAE + 93 mL/min/1.73 m²; *p* = 0.0079).

**Table 2:** KDIGO versus CTCAE eGFR_cr_ binary worsening endpoints

Interval	Endpoint	KDIGO+ /CTCAE+ (*n*=)	KDIGO+ /CTCAE− (*n*=)	KDIGO− /CTCAE+ (*n*=)	KDIGO− /CTCAE− (*n*=)	*p*
BL→FU	Any KDIGO eGFR_cr_ worsening	67	5	0	77	0.0625
LC→FU		54	5	0	87	0.0625
BL→FU	Clinically relevant KDIGO eGFR_cr_ worsening*	46	2	21	80	***< 0.0001***
LC→FU		36	2	18	90	***0.0002***

Baseline (BL) laboratory values prior to PRRT; eGFR_cr_ estimated glomerular filtration rate creatinine; Follow-up (FU) laboratory values at the last follow-up visit; Last cycle (LC) laboratory values after the last PRRT cycle; *defined as a decline in KDIGO category accompanied by a 25% or greater drop in eGFR_cr_ from baseline; + worsening; - no worsening; Significant values are marked in italic and bold

### eGFR_cr_ slope analysis differs across KDIGO/CTCAE discordance groups

Patients classified as nephrotoxic according to KDIGO or CTCAE showed significantly different annualized eGFR_cr_ slopes compared with patients without nephrotoxicity (KDIGO: median − 18.4 vs. −2.0 mL/min/1.73 m²/year, *p* < 0.0001; CTCAE: median − 12.8 vs. −1.1 mL/min/1.73 m²/year, *p* < 0.0001).

**Fig. 4:**
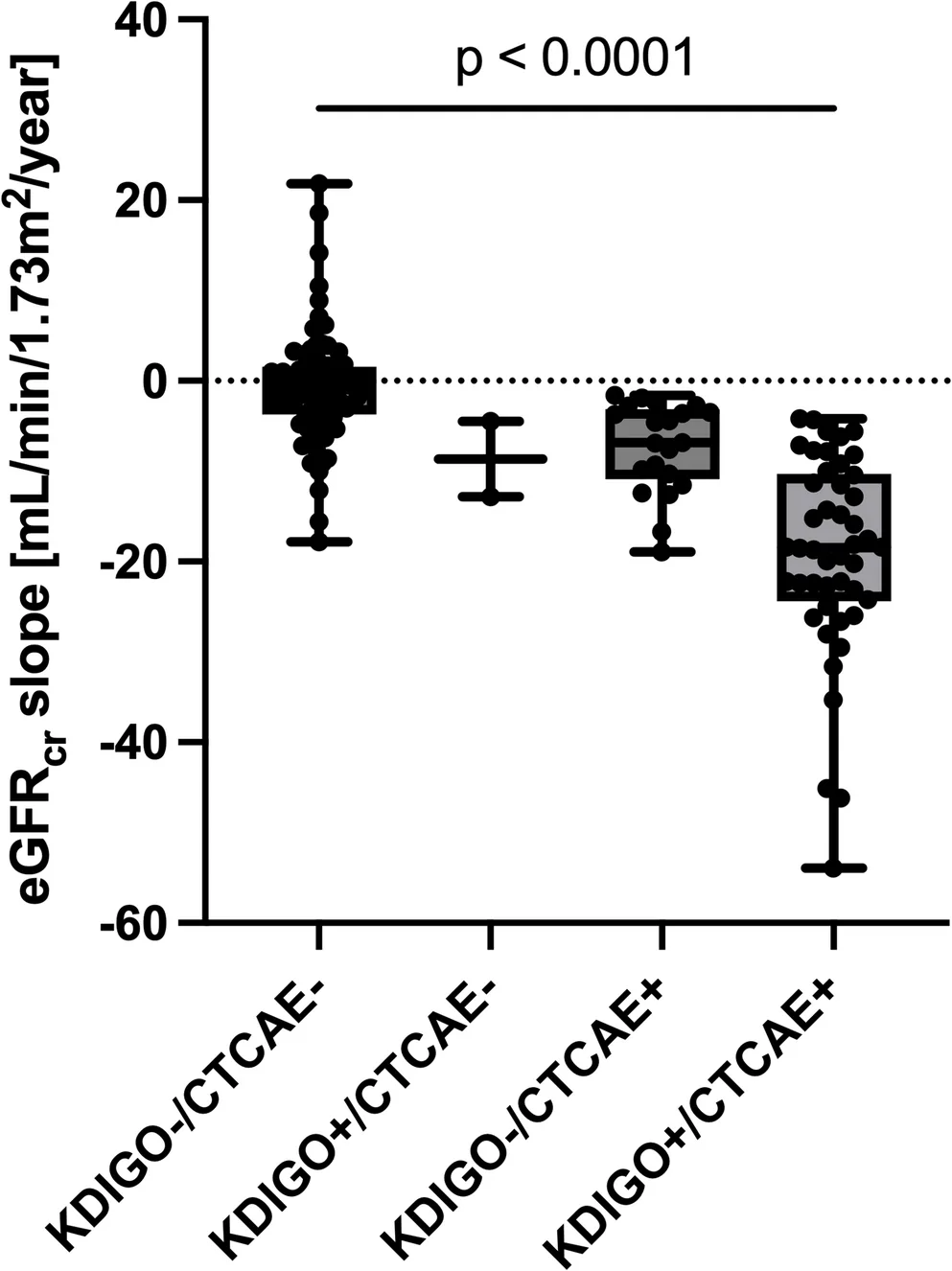
Boxplots demonstrating the eGFR slope across KDIGO/CTCAE discordance groups. Boxplots with individual data points show annualized eGFR_cr_ slope from LC to FU according to concordant and discordant KDIGO-derived and CTCAE-based eGFR_cr_ worsening groups. Median annualized eGFR_cr_ slope was − 0.95 in the KDIGO−/CTCAE− group, −8.65 in the KDIGO+/CTCAE− group, −6.8 in the KDIGO−/CTCAE+ group and − 18.45 in the KDIGO+/CTCAE+ group. Annualized eGFR slope differed significantly across KDIGO/CTCAE discordance groups (*p* < 0.0001). In post-hoc analysis, a significant difference was observed only for KDIGO−/CTCAE − vs. KDIGO+/CTCAE+ (*p* < 0.0001). Other pairwise comparisons did not reach statistical significance. BL, baseline; LC, last PRRT cycle; FU, last follow-up; eGFRcr, creatinine-based estimated glomerular filtration rate; CTCAE, Common Terminology Criteria for Adverse Events

Annualized eGFR_cr_ slope differed significantly across KDIGO/CTCAE discordance groups (*p* < 0.0001). Median annualized eGFR_cr_ slope was − 0.95 mL/min/1.73 m²/year in the KDIGO−/CTCAE− group, − 8.65 in the KDIGO+/CTCAE− group, − 6.8 in the KDIGO−/CTCAE+ group and − 18.45 in the KDIGO+/CTCAE+ group. Using post-hoc testing, a significant difference was observed only between KDIGO−/CTCAE − and KDIGO+/CTCAE+ patients (*p* < 0.0001). Other pairwise comparisons, including KDIGO+/CTCAE− versus KDIGO−/CTCAE−, did not reach statistical significance (see Fig. 4).

### Only CTCAE eGFR_cr_ worsening, but not KDIGO assessment from BL to LC is associated with eGFR_cr_ change during further FU

Renal classification changes from BL to LC were evaluated as a predictor of subsequent annualized eGFR_cr_ slope from the LC to FU. Absolute eGFR_cr_ at LC, but also both any and clinically relevant KDIGO eGFR category worsening were not significantly associated with subsequent eGFR_cr_ slope (*p* > 0.05). In contrast, only CTCAE eGFR_cr_ worsening from BL to LC was significantly associated with subsequent eGFR slope from LC to FU (β: −6.82; 95% CI: −10.06– −3.57; *p* < 0.0001; Table 3).

**Table 3:** Association of early KDIGO and CTCAE worsening from baseline (BL) to last cycle (LC) with subsequent eGFR_cr_ slope from last cycle (LC) to follow-up (FU)

Early predictor BL→LC	Outcome	β coefficient	95% CI	*p*-value
Any KDIGO eGFR_cr_ worsening	LC→FU eGFR_cr_ slope	0.22	−3.53 to 3.96	0.9088
Clinically relevant KDIGO eGFR_cr_ worsening		2.8	−3.57 to 7.74	0.4672
eGFR_cr_ at LC		−0.04	−0.13 to 0.05	0.3641
CTCAE eGFR_cr_ worsening		−6.82	−10.06 to −3.57	***< 0.0001***

Baseline (BL) laboratory values prior to PRRT; CI confidence interval; eGFR_cr_ estimated glomerular filtration rate creatinine; Follow-up (FU) laboratory values at the last follow-up visit; Last cycle (LC) laboratory values after the last PRRT cycle; Significant values are marked in italic and bold

Since follow-up duration was heterogeneous within the cohort, a stratified sensitivity analysis was performed according to follow-up duration. Patients were divided into short-term and longer-term follow-up subgroups using a 2-year cut-off (< 2 years vs. ≥2 years). CTCAE eGFR_cr_ worsening from BL to LC remained significantly associated with subsequent eGFR slope from LC to FU (< 2 years: β: −11.9; 95% CI: −17.66– −6.15; *p* = 0.0001; ≥ 2 years; β: −7.4; 95% CI: −9.93– −4.79; *p* < 0.0001). In a multiple linear regression model including CTCAE eGFR_cr_ worsening from BL to LC, follow-up duration group, and their interaction term, CTCAE eGFR_cr_ worsening remained significantly associated with subsequent eGFR_cr_ slope (β = −11.9; 95% CI, − 16.28 to − 7.54; *p* < 0.0001). Follow-up duration group itself was not significantly associated with eGFR_cr_ slope (β = −2.8; 95% CI, − 7.45 to 1.89; *p* = 0.2420). The interaction between CTCAE eGFR_cr_ worsening and follow-up duration group was not significant (β = 4.6; 95% CI, − 1.22 to 10.32; *p* = 0.1210).

### Comparison of eGFR calculations

Median eGFR_cys_ at BL was 70 mL/min/1.73 m² and median eGFR_cr/cys_ 79 mL/min/1.73 m² (eGFR_cys_ and eGFR_cr/cys_ pairs were available in *n* = 109 patients). Both eGFR_cys_ and eGFR_cr/cys_ decreased significantly to LC (Wilcoxon test: *p* < 0.0001, respectively). At both BL and LC eGFR_cys_ differed significantly from eGFR_cr_ and eGFR_cr/cys_ (all *p* < 0.0001), whereas eGFR_cr_ and eGFR_cr/cys_ did not differ significantly from each other (*p* > 0.05 each).

In exploratory regression analyses, early eGFR_cys_ loss from BL to LC was not significantly associated with subsequent annualized eGFR_cr_ slope from LC to FU (*p* = 0.1091). Early eGFR_cr/cys_ loss from BL to LC was significantly associated with subsequent annualized eGFR_cr_ slope (β 0.2197; *p* = 0.0072).

### Comparison of KDIGO 2012- and KDIGO 2024-derived eGFR_cr_ assessment

In a sensitivity analysis applying the KDIGO 2024-derived threshold of > 20% relative eGFR_cr_ decline, renal function deterioration from BL to FU was identified in 118 patients (79.2%), compared with 48 patients (32.2%) fulfilling the KDIGO 2012-derived clinically relevant worsening definition. From BL to LC, KDIGO 2024-derived > 20% eGFR_cr_ decline occurred in 100 patients (67.1%), compared with 13 patients (8.7%) fulfilling the KDIGO 2012-derived definition [8, 9].

When KDIGO 2024-derived eGFR_cr_ decline was compared with CTCAE eGFR_cr_ worsening, discordance was strongly asymmetric. For BL to FU, 67 patients were positive by both KDIGO 2024-derived and CTCAE criteria, 51 were KDIGO2024+/CTCAE−, none were KDIGO2024−/CTCAE+, and 31 were negative by both systems (exact McNemar test, *p* < 0.0001). For BL to LC, 51 patients were positive by both systems, 49 were KDIGO2024+/CTCAE−, 16 were KDIGO2024−/CTCAE+, and 33 were negative by both systems (exact McNemar test, *p* < 0.0001).

In prediction analysis, BL to LC KDIGO 2024-derived > 20% eGFR_cr_ decline was not associated with subsequent annualized eGFR_cr_ slope from LC to FU (β = 0.65; 95% CI, − 3.28 to 4.58; *p* = 0.7449).

### Risk-factor analysis

In univariable logistic regression analyses comparing early renal classification changes from BL to LC, age (OR 1.04 per year; 95% CI 1.00–1.07; *p* = 0.0239) and cumulative administered activity (OR 1.47 per 10 GBq; 95% CI 1.01–2.19; *p* = 0.0463) were found to be significantly associated with clinically relevant KDIGO eGFR_cr_ worsening. Sex, arterial hypertension and diabetes mellitus showed no significant association, see Table 4. In multivariable logistic regression, both age and cumulative administered activity remained significant (age: OR 1.04 per year; 95% confidence interval (CI) 1.01–1.08; *p* = 0.0125; cumulative administered activity/10 GBq: OR 1.64; 95% CI 1.08–2.48, *p* = 0.0205).

**Table 4:** Univariable risk factor analysis from baseline (BL) to last cycle (LC)

Endpoint	Predictor	Effect estimate	95% CI	*p*
Clinically relevant KDIGO eGFR_cr_ worsening	Age, per year	OR 1.04	1.00 to 1.07	***0.0239***
	Cumulative activity, per 10 GBq	OR 1.47	1.01 to 2.19	***0.0463***
	Sex	OR 0.64	0.32 to 1.28	0.2063
	Diabetes mellitus	OR 0.58	0.20 to 1.48	0.2610
	Arterial hypertension	OR 1.35	0.66 to 2.73	0.4090
CTCAE eGFR_cr_ worsening	Age, per year	OR 0.97	0.94 to 0.99	***0.0209***
	Cumulative activity, per 10 GBq	OR 1.22	0.84 to 1.79	0.2997
	Sex	OR 1.22	0.60 to 2.48	0.5860
	Diabetes mellitus	OR 0.59	0.20 to 1.53	0.2886
	Arterial hypertension	OR 0.72	0.34 to 1.45	0.3715

Baseline (BL) laboratory values prior to PRRT; CI confidence interval; eGFR_cr_ estimated glomerular filtration rate creatinine; Last cycle (LC) laboratory values after the last PRRT cycle; OR Odds Ratio; Significant values are marked in italic and bold

For CTCAE eGFR_cr_ worsening from BL to LC, age was the only significant predictor in univariable logistic regression, with higher age being associated with lower odds of recategorization (OR 0.97 per year; 95% CI 0.94–0.99; *p* = 0.0209), see Table 4.

## Discussion

Investigating long-term renal function following PRRT, we directly compared KDIGO- and CTCAE-based definitions of renal function deterioration. Relevant discordance was observed particularly when clinically relevant KDIGO eGFR_cr_ worsening was compared with CTCAE eGFR_cr_ worsening, indicating that both systems capture partly distinct aspects of renal deterioration. Of note, early CTCAE eGFR_cr_ worsening from BL to LC, but not KDIGO-based worsening, was associated with subsequent long-term eGFR_cr_ decline after completion of PRRT.

Our findings of gradual renal functional decline from BL to LC and FU are consistent with previous PRRT cohorts reporting gradual renal function loss following PRRT. For instance, Bergsma et al. reported an estimated annual decline in creatinine clearance of approximately 3.4% with no subacute CTCAE grade 3/4 nephrotoxicity and no patient showing an annual renal function decrease of ≥ 20% [16]. However, renal function was primarily assessed by creatinine clearance rather than eGFR_cr_ [16]. In a prospective dosimetry-guided [¹⁷⁷Lu]Lu-DOTATATE study, Sundlöv et al. reported a mean absolute annual measured GFR change of − 4.3 mL/min/1.73 m², which is close to the median annualized eGFRcr slope of − 4.5 mL/min/1.73 m²/year observed in our cohort, although their study used individualized kidney dosimetry, measured GFR, and a treatment strategy adapted to renal biologically effective dose [17]. Conversely, in a salvage PRRT cohort, Rudisile et al. reported no CTCAE grade 3/4 nephrotoxicity despite a higher absolute annual tubular extraction rate decrease of 8 ± 12 mL/min/1.73 m², highlighting that retreatment setting, prior therapies, cumulative treatment exposure, and the renal function metric used can substantially influence apparent nephrotoxicity rates [18]. They furthermore reported no severe nephrotoxicity in patients with kidney absorbed doses exceeding 23 Gy [18]. Subsequent studies and reviews have emphasized the generally favorable renal safety profile of ^177^Lu-based PRRT co-administered with renal protection, including in patients with preexisting chronic kidney disease [19–22]. More recent [¹⁷⁷Lu]Lu-DOTA-TATE data also support an overall favorable renal safety profile, with one cohort of 178 GEP-NET patients showing stable mean eGFR from baseline to follow-up and KDIGO-defined renal function decline in 8.9% of patients, with age emerging as the main predictor [23]. Taken together, the present median annualized eGFR_cr_ slope of − 4.5 mL/min/1.73 m²/year appears broadly consistent with the upper range of published longitudinal renal trajectories after [¹⁷⁷Lu]-based PRRT, but differences in baseline renal reserve, PRRT regimen, cumulative administered activity, use and composition of amino acid nephroprotection, patient-specific dosimetry, follow-up duration, and renal endpoint definition preclude direct one-to-one comparison.

Recent investigations have largely focused on CTCAE assessment from BL to FU [16, 20], whereas eGFR category-based frameworks derived from KDIGO may provide complementary information on renal function trajectories. However, because albuminuria was not available in the present cohort, our KDIGO-based analyses should be understood as eGFR_cr_ category-based assessments rather than complete KDIGO CKD staging. The observed discrepancy is rooted in the different conceptual frameworks of both systems: CTCAE is primarily designed to grade treatment-emergent adverse events in oncological trials, whereas KDIGO provides a nephrological framework for classifying kidney function and disease progression [7, 8].

The high weighted kappa values indicate that KDIGO and CTCAE GFR-based classifications show very good overall agreement when considered as ordinal grading systems. Nevertheless, the gradual decrease in weighted kappa from baseline to last follow-up suggests that classification discordance increases over time. Importantly, high overall agreement does not exclude clinically relevant differences in toxicity detection, as kappa is strongly influenced by the large proportion of concordantly classified patients. In this regard, we observed that nephrotoxicity classification after PRRT depends substantially on the applied toxicity framework and on the definition of renal deterioration. When any KDIGO eGFR_cr_ category worsening was considered, classification did not differ significantly from CTCAE eGFR_cr_-based grading. This is supported by the McNemar analyses, which showed significant discordance when KDIGO worsening was restricted to clinically relevant GFR deterioration. This suggests that broad KDIGO category-based worsening captures minor threshold-crossing changes, whereas clinically relevant KDIGO worsening may define a more restrictive endpoint. Both KDIGO assessments, however, seem not to be fully interchangeable with CTCAE grading.

The asymmetry of discordant cases further illustrates the conceptual differences between both classification systems. In the comparison of clinically relevant KDIGO-derived eGFR_cr_ worsening and CTCAE eGFR_cr_ worsening from BL to FU, substantially more patients were classified as KDIGO−/CTCAE+ than KDIGO+/CTCAE−. Baseline renal function differed significantly between these discordant subgroups (*p* = 0.0079). This suggests that clinically relevant KDIGO-derived worsening preferentially identifies patients with lower baseline renal reserve in whom deterioration fulfills both category worsening and relative decline criteria. Conversely, CTCAE grading may capture milder renal function changes in patients with higher baseline eGFR_cr_ that do not meet the more restrictive KDIGO-derived definition of clinically relevant worsening. This baseline-dependent behavior may explain the observed imbalance between discordant subgroups and reinforces that CTCAE and KDIGO-derived endpoints should not be used interchangeably.

Annualized eGFR_cr_ slope provided a complementary quantitative measure of renal function change. Patients classified as nephrotoxic by both KDIGO and CTCAE showed the steepest eGFR_cr_ decline, while KDIGO-only cases did not differ significantly from patients without nephrotoxicity after post-hoc testing. Therefore, the slope analysis supports the internal consistency of the classification systems but should not be interpreted as an independent validation, since eGFR_cr_ slope and nephrotoxicity categories are derived from overlapping renal function data.

The KDIGO 2024-derived sensitivity analysis further demonstrated that endpoint sensitivity does not necessarily translate into predictive utility. The > 20% eGFR_cr_ decline threshold identified substantially more patients than the stricter KDIGO 2012-derived definition; however, BL to LC KDIGO 2024-derived decline was not associated with subsequent annualized eGFR_cr_ slope.

In exploratory cystatin C-based analyses, both eGFR_cys_ and eGFR_cr/cys_ decreased significantly from BL to LC, supporting the feasibility of incorporating cystatin-based renal function estimates into PRRT nephrotoxicity monitoring. However, although early eGFR_cr/cys_ loss was associated with subsequent eGFR_cr_ slope, the direction of this association did not indicate accelerated further renal decline; therefore, these subgroup findings should be interpreted as exploratory and require prospective validation.

The exploratory prediction analysis provides an additional clinically relevant perspective. Early renal classification changes from BL to LC were tested as predictors of subsequent annualized eGFR_cr_ slope from the last cycle to long-term follow-up. Neither *any* KDIGO eGFR_cr_ category worsening nor *clinically relevant* KDIGO worsening was significantly associated with subsequent eGFR slope. Absolute eGFR_cr_ at the last PRRT cycle was also not predictive for long-term renal outcome, thereby highlighting the need for a more sophisticated approach to identify high-risk patients at this particular time-point of treatment completion. In this regard, early CTCAE eGFR_cr_ worsening from baseline to the last cycle was strongly associated with subsequent eGFR decline. Because follow-up duration varied substantially, we additionally examined whether the association between early CTCAE eGFR_cr_ worsening and subsequent renal decline was preserved across follow-up strata. In this sensitivity analysis, early CTCAE eGFR_cr_ worsening from BL to LC remained significantly associated with subsequent annualized eGFR_cr_ slope in both short-term and longer-term follow-up subgroups. This suggests that changes determined by this particular framework may be more useful for identifying patients requiring closer renal surveillance after completion of PRRT. However, this analysis was exploratory and should be validated prospectively before being used for treatment or follow-up decisions.

Nonetheless, the herein presented data on the predictive value of BL to LC classification changes for long-term renal outcome are of relevance, as current guidelines recommend a minimum of four treatment cycles and thus, there is a clinical need to provide robust long-term outcome predictors after completion of PRRT [5]. Neglecting changes of renal function under PRRT, however, may inherit the risk to miss subjects experiencing relevant eGFR decline, as a simple evaluation of absolute eGFR_cr_ values at this time point failed to reach significance in outcome analysis. These considerations are further supported by our risk-factor analysis showing an association between cumulative administered activity and clinically relevant KDIGO-derived eGFR_cr_ worsening. However, cumulative administered activity should not be interpreted as equivalent to renal radiation burden. It is only an indirect treatment-exposure parameter and does not account for patient-specific pharmacokinetics, renal tracer handling, kidney volume, baseline renal reserve, amino acid protection, or the actual absorbed dose to renal parenchyma. Because patient-level renal dosimetry was not performed, the causal link between radiation exposure and progressive renal function decline cannot be established in the present study. Therefore, the observed association should be interpreted as hypothesis-generating rather than causal and should ideally be evaluated in future studies incorporating post-therapeutic dosimetry assessments.

The additional observed association with age is also clinically plausible, as older patients may be more likely to cross clinically relevant eGFR_cr_ thresholds even with comparable absolute renal function loss [24]. In previous studies, age was also demonstrated as predictor of renal function decline [23]. In contrast, arterial hypertension, diabetes mellitus and sex were not significantly associated with renal deterioration in this cohort, which is in line with previous data showing no clear association with those parameters [3, 16]. Differences between studies may reflect variation in endpoints, follow-up duration, baseline renal reserve, radionuclides, renal protection protocols and how renal function had been determined.

Several limitations need to be acknowledged. This was a retrospective single-center analysis, and laboratory testing was not performed according to a prospective nephrotoxicity protocol. KDIGO was applied as an eGFR_cr_-based classification only. The full KDIGO CKD framework classifies kidney disease by cause, eGFR_cr_ category and albuminuria category, but albuminuria was not routinely assessed in our clinical workflow and was therefore unavailable for analysis [8, 9]. This limitation may have biased renal deterioration classification in several ways, since patients with stable or only mildly declining eGFR_cr_ but new or worsening albuminuria could not be identified, potentially underestimating clinically relevant kidney injury. Furthermore, albuminuric progression, which may reflect glomerular damage, endothelial dysfunction, or radiation-associated renal microvascular injury, was not captured by our eGFR-only endpoint [25–27]. Patients with the same eGFR_cr_ category may have substantially different nephrological risk profiles depending on albuminuria status; therefore, our analysis could not reproduce KDIGO risk stratification as used in real-world nephrology workflows [8, 26]. Conversely, eGFR_cr_ category worsening without albuminuria information may overemphasize threshold-crossing changes in filtration function that are not necessarily accompanied by broader evidence of kidney disease. Accordingly, our findings should be interpreted as PRRT-associated eGFR_cr_ trajectory analyses using KDIGO-derived category thresholds, rather than as full KDIGO nephrotoxicity staging. Additionally, the present analysis used the KDIGO 2012 definition of clinically relevant eGFR_cr_ worsening rather than the 2024 practice point of > 20% eGFR_cr_ change [8, 9]. This choice was made because KDIGO 2012 provides a clear progression endpoint defined by eGFR category worsening plus a ≥ 25% eGFR decline from baseline [8, 9]. Nonetheless, the underlying KDIGO eGFR categories remained unchanged between the 2012 and 2024 guidelines and thus, ordinal KDIGO eGFR_cr_ classification was not affected by this choice [8, 9]. Furthermore, direct GFR measurement, for example by iohexol plasma clearance, would provide a more robust assessment of renal function in this setting [28]. Patient-level renal dosimetry was not available in the present cohort. Therefore, cumulative administered activity was used only as a treatment-exposure surrogate and cannot be considered a direct measure of renal radiation burden. Concomitant medication represents an additional potential confounder that could not be fully addressed in the present retrospective analysis. Kidney function estimates based on serum creatinine may be influenced by medication-related changes in renal hemodynamics, volume status, muscle metabolism, changes in body weight, muscle mass, and nutritional status. Because detailed longitudinal medication exposure was not systematically available, residual confounding by concomitant medication cannot be excluded. No clinically relevant body weight changes were identified during PRRT. Nevertheless, because body weight and body composition were not assessed according to a standardized prospective protocol, residual influence of changes in muscle mass or nutritional status on creatinine-based eGFR estimates cannot be fully excluded.

Taken together, future studies should integrate renal dosimetry, and measured GFR, albuminuria assessment, structured recording of nephroprotective and nephrotoxic co-medication and prospective renal follow-up.

## Conclusion

In conclusion, KDIGO-derived and CTCAE-based eGFR_cr_ classifications provide complementary but non-interchangeable information on renal function changes after PRRT. Clinically relevant KDIGO-derived eGFR_cr_ worsening represents a stricter filtration-based endpoint, whereas early CTCAE eGFR_cr_ worsening from BL to LC was associated with subsequent long-term eGFR_cr_ decline and may help identify patients requiring closer post-treatment renal surveillance. Patients with early CTCAE-based eGFR_cr_ worsening may therefore benefit from risk-adapted surveillance, including repeat eGFR_cr_ testing within 3 months after PRRT completion, renal monitoring every 3–6 months during the first year, as well as optimization of modifiable renal risk factors, and individualized renal dosimetry before further PRRT. Prospective studies combining serial filtration markers, urine biomarkers, and individualized kidney dosimetry are needed to validate renal risk stratification after PRRT, particularly as PRRT is increasingly being evaluated in earlier disease settings.

## Supplementary Information

Below is the link to the electronic supplementary material.

Supplementary Material 1 (DOCX 13.6 KB)

## Data Availability

The datasets used and/or analysed during the current study are available from the corresponding author on reasonable request.

## Abbreviations

BL: labs at baseline
CI: confidence interval
CrCl: creatinine clearance
CTCAE: Common Terminology Criteria for Adverse Events
eGFR_cr_: estimated glomerular filtration rate creatinine
eGFR_cys_: estimated glomerular filtration rate cystatin c
eGFR_cr/cys_: estimated glomerular filtration rate creatinine/cystatin c
FU: labs at the last follow-up visit
GEP-NET: gastroenteropancreatic neuroendocrine tumors
IQR: interquartile range
KDIGO: Kidney Disease: Improving Global Outcomes
LC: labs after the last PRRT cycle
PRRT: peptide receptor radionuclide therapy
OR: odds ratio
