# Supplementary material for: Comparative assessment of KDIGO and CTCAE criteria for detecting renal function deterioration after peptide receptor radionuclide therapy
Source: Eur J Nucl Med Mol Imaging. 2026 Aug 12;53(13):8272–83. doi: 10.1007/s00259-026-08126-4 (PMC13727837; doi:10.1007/s00259-026-08126-4)
Supplement: Supplementary file 1 [file 259_2026_8126_MOESM1_ESM.docx]

**SUPPLEMENTS**

**Supplemental Table 1: Distribution of renal classifications**

| **Classification** | **Category/grade** | **BL (n=)** | **LC (n=)** | **FU (n=)** |
| --- | --- | --- | --- | --- |
| KDIGO eGFR_cr_ | G1 | 65 | 48 | 32 |
|  | G2 | 65 | 77 | 69 |
|  | G3a | 17 | 15 | 18 |
|  | G3b | 2 | 6 | 18 |
|  | G4 | 0 | 0 | 10 |
|  | G5 | 0 | 0 | 2 |
| CTCAE eGFR_cr_ | 0 | 65 | 48 | 32 |
|  | 1 | 65 | 77 | 69 |
|  | 2 | 19 | 21 | 36 |
|  | 3 | 0 | 0 | 10 |
|  | 4 | 0 | 0 | 2 |

BL labs at baseline; eGFR_cr_ estimated glomerular filtration rate creatinine; FU labs at the last follow-up visit; LC labs after the last PRRT cycle
